# Evaluating Personalized (N-of-1) Trials in Rare Diseases: How Much Experimentation Is Enough?

**DOI:** 10.1162/99608f92.e11adff0

**Published:** 2022-09-08

**Authors:** Ken Cheung, Hiroshi Mitsumoto

**Affiliations:** 1 Mailman School of Public Health, Columbia University, New York City, New York, United States of America; 2 Columbia University Irving Medical Center, Columbia University, New York City, New York, United States of America

**Keywords:** ALS, heterogeneity of treatment effects (HTE), minimally clinically important heterogeneity, patient-centered research, rare diseases, sample size formulae

## Abstract

For rare diseases, conducting large, randomized trials of new treatments can be infeasible due to limited sample size, and it may answer the wrong scientific questions due to heterogeneity of treatment effects. Personalized (N-of-1) trials are multi-period crossover studies that aim to estimate individual treatment effects, thereby identifying the optimal treatments for individuals. This article examines the statistical design issues of evaluating a personalized (N-of-1) treatment program in people with amyotrophic lateral sclerosis (ALS). We propose an evaluation framework based on an analytical model for longitudinal data observed in a personalized trial. Under this framework, we address two design parameters: length of experimentation in each trial and number of trials needed. For the former, we consider patient-centric design criteria that aim to maximize the benefits of enrolled patients. Using theoretical investigation and numerical studies, we demonstrate that, from a patient’s perspective, the duration of an experimentation period should be no longer than one-third of the entire follow-up period of the trial. For the latter, we provide analytical formulae to calculate the power for testing quality improvement due to personalized trials in a randomized evaluation program and hence determine the required number of trials needed for the program. We apply our theoretical results to design an evaluation program for ALS treatments informed by pilot data and show that the length of experimentation has a small impact on power relative to other factors such as the degree of heterogeneity of treatment effects.

## 1. Introduction

When managing chronic diseases and conditions, patients commonly try different treatments over time before finding the right treatments. The practice of N-of-1 trials operationalizes this type of patient-centered experimentation by randomizing treatments to single patients in multiple crossover periods, often in a balanced fashion. N-of-1 trials can be used to identify the optimal personalized treatment for single patients in situations involving evidence for heterogeneity of treatment effects (HTE) or the lack of a cure (Davidson et al. 2021). As such, these trials are sometimes called single-patient trials or personalized trials. First introduced by (Hogben and Sim 1953), N-of-1 trials have recently been applied to treat rare diseases (Roustit et al. 2018), as well as common chronic conditions such as hypertension (Kronish et al. 2019)(Samuel et al. 2019). The use of personalized (N-of-1) trials in treating rare diseases is particularly appealing because demonstrating comparative effectiveness of treatments at the population level via parallel-group randomized trials is often infeasible.

In this article, we consider personalized (N-of-1) trials of treatments for people with amyotrophic lateral sclerosis (ALS). ALS is a rare neurodegenerative disease that affects motor neurons in the brain and spinal cord. Despite the fact that two modestly effective disease-modifying medications have been approved for the treatment of ALS (Edaravone [MCI-186] ALS 19 Study Group 2017), the disease has no cure, and thus, symptomatic treatments remain an important strategy to improve the quality of life in people with ALS (Mitsumoto, Brooks, and Silani 2014). In particular, muscle cramps are disabling symptoms affecting over 90% of ALS patients, with demonstrated between-patient variability and yet stable manifestation of symptoms in a patient (Caress et al. 2016). Several treatments targeting muscle cramps have been evaluated and have shown mixed results, suggesting the presence of HTE or inadequate statistical power for definitive conclusions (Baldinger, Katzberg, and Weber 2012). Furthermore, ALS itself has been considered markedly heterogeneous in its pathogeneses, disease manifestations, and disease progression (Al-Chalabi and Hardiman 2013)(van den Berg et al. 2019). These are the clinical situations in which personalized (N-of-1) trials can help patients identify the best treatments for themselves (n.d.a).

Despite renewed interest in N-of-1 trials and numerous recent applications, the literature has offered little discussion on the evaluation of the usefulness of N-of-1 trials. As N-of-1 trials typically require active physician involvement, intense monitoring, and frequent data collection compared with usual care, these additional costs and resources warrant careful evaluation of effectiveness before said trials are used in practice as regular clinical service. The primary evaluation question is “Does the practice of N-of-1 trials in clinical care improve outcomes on the standard of care?” However, presuming the quality of treatment decisions based on N-of-1 trials is higher than what standard of care would prescribe, reports of N-of-1 trials often describe only the applications and results of the trials without plans to address the evaluation question. An exception is (Kravitz et al. 2018) who compare N-of-1 intervention against the usual care for patients with musculoskeletal pain in a randomized fashion using data collected after experimentation ends and find no evidence of superior outcomes among participants undergoing N-of-1 trials. However, when planning the study, the authors had not considered the underlying model that accounts for variability and correlation in the longitudinal observations and the assumptions on the effect size, which would in turn drive the appropriate sample size of an evaluation program for N-of-1 trials. A design issue related to sample size determination is the duration of experimentation in N-of-1 trials. In this article, we propose a framework to evaluate the quality and effectiveness of N-of-1 trials and develop specific guidance to address these design issues. We will introduce the evaluation framework in Section 2 and define the basic analytical model for analyzing N-of-1 trials in Section 3. The main findings on the experimentation duration and sample size are derived and described in Section 4 and applied to the ALS treatment program in Section 5. The article ends with a discussion in Section 6. All technical details are provided in the Appendices.

## 2. An Evaluation Framework for Personalized (N-of-1) Trials

### 2.1. The Anatomy of a Personalized (N-of-1) Trial

**Figure 1.**
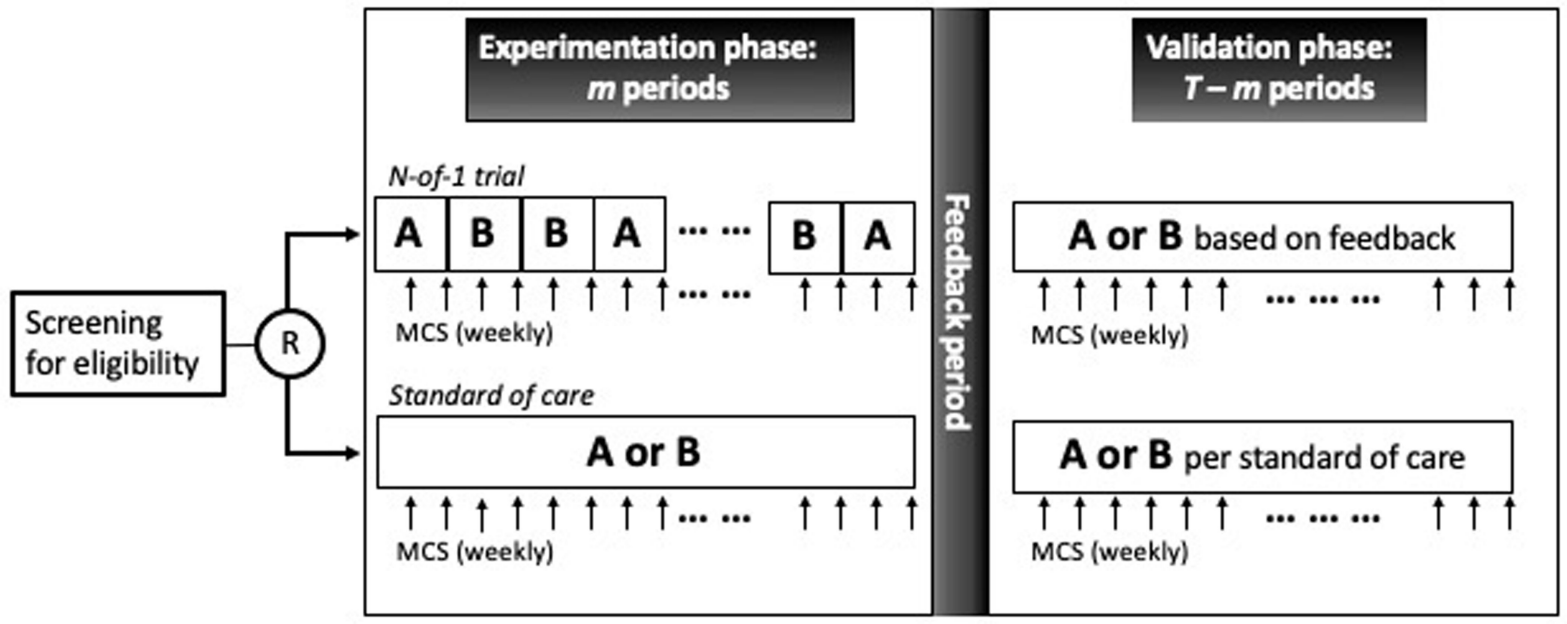
Schema of an evaluation program for personalized (N-of-1) trials comparing treatment A and treatment B. Under the evaluation program, patients are randomized to either an N-of-1 trial or the standard of care.

We consider an evaluation program comparing the effectiveness of personalized (N-of-1) trials in treating muscle cramps in people with ALS relative to the institutional standard of care. Under the program, people with ALS will be randomized to receive personalized (N-of-1) trials that compare two standard drugs prescribed for muscle cramps, mexiletine and baclofen. In each trial, a patient will be given the two drugs sequentially over T=18 two-week treatment periods in two phases. The first phase consists of m treatment periods (with m<T) when the two drugs are randomized in a multiple crossover fashion. This phase shall be referred to as the experimentation phase. In the remaining T−m treatment periods, the patient will continue with a drug treatment selected based on data in the experimentation phase. This phase shall be referred to as the validation phase (Figure 1).

During the treatment periods, the Columbia Muscle Cramp Scale (MCS) will be collected weekly to result in two MCS measurements for each period: one at the end of week 1 and one at the end of week 2. The MCS is a validated, composite score summarizing the frequency, severity, and clinical relevance of cramps in people with ALS (Mitsumoto et al. 2019). While the study does not include washout periods between treatments, only the measurement at the end of each two-week period will be used in the primary analysis in order to avoid carryover effects of the drugs.

Sandwiched between the two treatment phases is a feedback period where the MCS data in the experimentation phase are reviewed with the treating physician and the patient. The feedback period enables data-driven treatment decisions by providing the stakeholders with data visualization as well as numerical comparison (Davidson et al. 2021).

### 2.2. Standard of Care

In this article, we focus on a randomized controlled evaluation program where patients are randomized between an N-of-1 trial and standard of care (SOC). As depicted in Figure 1, a patient under SOC will be given either mexiletine or baclofen for 36 weeks, corresponding to the 18 two-week treatment periods in the N-of-1 trials, and will have the same follow-up schedule as the N-of-1 trial patients. Treatments in the ‘experimentation phase’ will be determined by the treating physicians. The ‘feedback period’ in the SOC arm may be viewed as a sham intervention and be conducted as a regular clinic visit before the patient continues into the ‘validation phase’ with the same drug in the remaining T−m treatment periods. By the virtue of randomization, MCS collected in the validation phase under SOC will serve as the control data and allow for an unbiased comparison with the validation phase in the N-of-1 trial patients.

Let $p_{0}$ denote the probability that mexiletine will be prescribed under SOC and $p_{1}$ the probability baclofen will be prescribed such that $p_{0}+p_{1}=1$. The special case $p_{0}=1$ and $p_{1}=0$ corresponds to a clinical scenario where mexiletine is considered the standard treatment. Generally, the program probability parameters $p_{0},p_{1}$ are somewhere between 0 and 1 when no clear best treatment exists. A program equipoise may be defined as when the treating physicians will give either of the drugs with equal likelihood, that is, $p_{0}=p_{1}=0.5$. These program parameters apparently affect the quality of treatment under standard of care, and hence the advantage of N-of-1 trials over standard of care. At the end of the evaluation program, these parameters can be estimated using the control data.

### 2.3. Design Parameters

While the study duration (or the number of treatment periods T) is determined based on feasibility and how long a patient can be followed in the evaluation program, an N-of-1 trial under the evaluation framework is defined by the length m of the experimentation phase, and hence the length T−m of the validation phase. Intuitively, the quality of the treatment decision by an N-of-1 trial improves with a larger m as more data will be available during the feedback period. On the other hand, a long experimentation phase may place excessive burden on patients without benefitting them and imply a short validation phase for a given T. Rather than maximize accuracy, the experimentation length m will respond to the question “How much experimentation is needed for an N-of-1 trial to be beneficial to an individual?”

A second design parameter is the specification of an analytical plan used to guide treatment selection during the feedback period. Principled statistical or data science methods should be employed to ensure the analysis is rigorous, while a prespecified plan entails preprogrammed algorithms that in turn facilitate quick feedback to the stakeholders.

Finally, as in conventional randomized controlled trials, the number of patients randomized in an evaluation program will need to be determined to ensure adequate statistical power for the primary evaluation question on whether N-of-1 trials improve outcomes.

To summarize, the design parameters that need to be prespecified at the planning stage of an evaluation program are the primary analysis plan used in the feedback period, the experimentation length (m) for each individual, and the number of individuals required. These will be discussed in next two sections.

## 3. An Analytical Model for N-of-1 Trials

Let $y_{it}$ be the outcome of patient i in treatment period t and $x_{it}\in \{-1,1\}$ be the corresponding treatment for i=1,…,n and t=1,…,T. Without loss of generality, we assume a large value of the outcome $y_{it}$ is desirable. To put the notation in the context of our study, we let $y_{it}$ denote the negative value of MCS at the end of each two-week treatment period. For the treatments, baclofen is coded as $x_{it}=1$ and mexiletine as $x_{it}=-1$. In this article, we focus on balanced sequences between baclofen and mexiletine in the experimentation phase, that is, assuming

(3.1) $$\sum_{t=1}^{m}x_{it}=0.$$

Consider the outcome model

(3.2) $$y_{it}=\alpha_{i}+\beta_{i}x_{it}+\epsilon_{it}$$

where $\beta_{i}$ is the patient-specific treatment effect and the noise $\epsilon_{it}\text{s}$ are mean zero normal with cov $(\epsilon_{it},\epsilon_{is})=\rho_{st}\sigma^{2}$ and $\rho_{tt}=1$. To reflect heterogeneous symptoms and HTE among the patients, we postulate $\alpha_{i}∼N(\mu_{A},\sigma_{A}^{2})$ and $\beta_{i}∼N(\mu_{B},\sigma_{B}^{2})$. The mean $\mu_{B}$ indicates the average treatment effect and the variance $\sigma_{B}^{2}$ indicates the extent of HTE in the disease population. While $\mu_{B}=0$ represents the null scenarios where there is no average treatment effects, a large value of $\sigma_{B}^{2}$ indicates the needs for personalizing treatments.

Under model (3.2), the optimal treatment for patient i can be expressed as $2I(\beta_{i}>0)-1$, where I(⋅) is an indicator function. During the feedback period, we may present to patient i an estimated treatment effect ${\hat{\beta }}_{i}$ based on the experimentation phase data $\left\{(x_{it},y_{it}):t=1,\ldots ,m\right\}$ along with the estimated optimal personalized treatment for the patient:

(3.3) $$x_{i}^{*}=2I({\hat{\beta }}_{i}>0)-1.$$

Subsequently, in the event of perfect adherence to analysis result, the patient will receive the estimated optimal treatment (3.3) in the validation phase, that is, $x_{it}\equiv x_{i}^{*}$ for t=m+1,…,T.

Some practical notes on the choice of ${\hat{\beta }}_{i}$ are in order. For the purposes of providing quick feedback, a broad range of estimators can be considered. The theoretical results derived in the following sections will hold for any estimators that are approximately normally distributed with mean $\beta_{i}$ and some finite variance $\tau_{i}^{2}$. A simple example is the the patient-specific least squares estimator ${\hat{\beta }}_{i}^{LS}=\sum_{t=1}^{m}x_{it}y_{it}/m$ for patient i. The least squares estimator is unbiased for the patient-specific treatment effect $\beta_{i}$ regardless of the variance-covariance structure of {ϵ} with variance

(3.4) $$\tau_{i}^{2}=\text{var}({\hat{\beta }}_{i}^{LS}|\alpha_{i},\beta_{i})=\lambda_{i}\sigma^{2}/m\;\text{where}\;\lambda_{i}=1+\sum_{s\neq t}x_{is}x_{it}\rho_{st}/m.$$

Note that the conditional variance (3.4) is free of the patient-specific parameters $\alpha_{i}$ and $\beta_{i}$. For the purposes of planning an N-of-1 trial, we will focus on the use of least squares. However, in the actual analysis, if additional information is available to inform the appropriate correlation structure of the data, likelihood-based estimation or weighted least squares accounting for such structure may improve efficiency.

## 4. How Much Experimentation Is Enough?

### 4.1. Patient-Centric Criteria and Length of Experimentation Phase

In this subsection, we discuss the choice of the experimentation length m of an N-of-1 trial with respect to two different patient-centric criteria, both of which aim to maximize the benefits to patients on N-of-1 trials.

The first criterion is defined as the expected number of periods where a patient receives the optimal treatment. Mathematically, this criterion is denoted as $E(z_{i})$, where $z_{i}$ is the number of periods in which patient i receives the optimal treatment over the T treatment periods.

#### Proposition 1.

*Suppose* ${\hat{\beta }}_{i}∼N(\beta_{i},\tau_{i}^{2})$ *under a balanced experimentation phase (3.1). Then for* 0<m≤T,

$$E(z_{i})=\frac{m}{2}+(T-m)\;\text{Pr}(W\leq \frac{\left|\mu_{B}+\sigma_{B}U\right|}{\tau_{i}})$$

*where* W,U *are independent standard normal variables. Furthermore, if* $\mu_{B}=0$, *then*

(4.1) $$E(z_{i})=\frac{m}{2}+(T-m)\;G(\sigma_{B}/\tau_{i})$$

*where* G *is the cumulative distribution function of* W/|U|, which is a pivotal distribution.

The second patient-centric criterion is defined as the expected average outcome of a patient during an N-of-1 trial. This criterion is denoted as $E({\overset{¯}{y}}_{i})$, where ${\overset{¯}{y}}_{i}=\sum_{t=1}^{T}y_{it}/T$ is the average outcome of the patient in all T treatment periods.

#### Proposition 2.

*Under the same condition as in Proposition 1, for* 0<m≤T,

$$E({\overset{¯}{y}}_{i})=\mu_{A}+(1-\frac{m}{T})[\mu_{B}\{2\Phi (\frac{\mu_{B}}{\sqrt{\sigma_{B}^{2}+\tau_{i}^{2}}})-1\}+\frac{2\sigma_{B}^{2}}{\sqrt{\sigma_{B}^{2}+\tau_{i}^{2}}}\phi (\frac{\mu_{B}}{\sqrt{\sigma_{B}^{2}+\tau_{i}^{2}}})]$$

*where* Φ *and* ϕ *respectively denote the standard normal distribution function and density*.

We can derive a few practical principles from Proposition 1 and Proposition 2. First, conducting an N-of-1 trial with an experimentation length m<T is generally beneficial for the patient compared to experimentation in all T period. Specifically, we can derive from Proposition 1 that the patient will receive at least half of the time, that is, $E(z_{i})\geq T/2$ for all m, and attain the minimum when m=T. Analogously from Proposition 2, the expected average outcome will be no smaller than the population average, that is, $E({\overset{¯}{y}}_{i})\geq \mu_{A}$ for all m, and equality holds when m=T.

Second, we can derive from the propositions that $E(z_{i})$ and $E({\overset{¯}{y}}_{i})$ are increasing in $\sigma_{B}$ under the null $\mu_{B}=0$. In other words, an N-of-1 trial becomes more beneficial to the patient when there is a larger variability in the treatment effects across patients.

Third, and importantly, considering the null case where $\mu_{B}=0$ and when the least squares ${\hat{\beta }}_{i}^{LS}$ is used to make inference based on the experimentation phase data is instructive. Under these conditions, we can derive from Proposition 2 that the criterion $E({\overset{¯}{y}}_{i})$ is maximized at

(4.2) $$m^{*}=\frac{2T}{\sqrt{9+8\xi_{i}T}+3}$$

where $\xi_{i}=\frac{\sigma_{B}^{2}}{\lambda_{i}\sigma^{2}}$ and $\lambda_{i}$ is defined in (3.4). While Equation (4.2) gives the optimal length $m^{*}$ as a function $\sigma_{B},\sigma ,$ and $\lambda_{i}$, it provides some general guidance:

#### Main Result 1.

The optimal experimentation length $m^{*}$ is less than one-third of the total N-of-1 trial duration from a patient’s perspective, that is, $m^{*}≲T/3$.

### 4.2. Sample Size

In this subsection, we discuss how much experimentation is adequate in terms of the sample size enrolled to the evaluation program. We first define the quality of an N-of-1 trial as the expected health outcome under the estimated optimal treatment $x_{i}^{*}$ (3.3). Assuming perfect adherence to the analysis results in the feedback period, the quality of an N-of-1 trial can be defined as $E(y_{i}^{*})$ where $y_{i}^{*}=\sum_{t=m+1}^{T}y_{it}/(T-m)$ and the expectation is taken with respect to the distributions of $\alpha_{i},\beta_{i},\;x_{i}^{*}$, and $\left\{\epsilon_{it}\right\}$. Analogously, we can define the quality of standard of care as $E(y_{i}\prime )$ where $y_{i}\prime$ is the average outcome observed in the validation phase for a patient under SOC and the expectation is taken under the assumption that the treatment in the experimentation phase continues to the validation phase. The primary objective of the evaluation program is to compare the quality of an N-of-1 trial and the quality of SOC. This can be formulated into a hypothesis testing problem with

(4.3) $$H_{0}:\Delta :=E(y_{i}^{*})-E(y_{i}\prime )\leq 0\;\text{versus}\;H_{1}:\Delta >0$$

where Δ measures the degree of quality improvement due to N-of-1 trials defined over the patient population. The hypotheses (4.3) can in turn be tested using the regular Z-statistic:

(4.4) $$Z=\frac{\sqrt{n}({\overset{¯}{y}}^{*}-\overset{¯}{y}\prime )}{\sqrt{v^{*}+v\prime }}$$

where ${\overset{¯}{y}}^{*}$ and $v^{*}$ are respectively the sample mean and the sample variance of $\left\{y_{i}^{*}\right\}$ in the n patients randomized to an N-of-1 trial and $\overset{¯}{y}\prime$ and v′ are the sample mean and sample variance of $\left\{y_{i}\prime \right\}$ in the n patients randomized to SOC. Using standard arguments gives the power of the Z-test

(4.5) $$\text{Pr}(Z>c_{\alpha }|\Delta )\approx \Phi (\frac{\sqrt{n}\Delta }{\sqrt{\text{var}(y_{i}^{*})+\text{var}(y_{i}\prime )}}-c_{\alpha })$$

where $c_{\alpha }$ is the upper αth percentile of standard normal. In Appendix C, we derive the expressions for Δ, var $\left(y_{i}^{*}\right)$, and var $\left(y_{i}\prime \right)$ in (4.5) under the condition that $\tau_{i}^{2}\equiv \tau^{2}$ for all i. This condition is met when the N-of-1 trial patients receive the same sequence $x_{it}$ in the experimentation phase or when $\left\{\epsilon_{it}\right\}$ has a specific variance-covariance structure. For example, under a compound symmetry, that is, $\rho_{st}\equiv \rho$, we can show $\lambda_{i}\equiv \lambda =1-\rho$, that is, having $\tau_{i}^{2}\equiv (1-\rho )\sigma^{2}/m$ for all i. Specifically, under the assumption that the SOC treatment $x_{i}\prime$ for a given patient is independent of the patient-specific treatment effect $\beta_{i}$, we have

(4.6) $$\Delta =2\mu_{B}\{\Phi (\frac{\mu_{B}}{\sqrt{\sigma_{B}^{2}+\tau_{i}^{2}}})-p_{1}\}+\frac{2\sigma_{B}^{2}}{\sqrt{\sigma_{B}^{2}+\tau_{i}^{2}}}\phi (\frac{\mu_{B}}{\sqrt{\sigma_{B}^{2}+\tau_{i}^{2}}}),$$

(4.7) $$\text{var}(y_{i}^{*})=\sigma_{A}^{2}+\sigma_{B}^{2}+\mu_{B}^{2}-\{\Delta +\mu_{B}(2p_{1}-1){\}}^{2}+\frac{\sigma^{2}}{T-m},$$

and

(4.8) $$\text{var}(y_{i}\prime )=\sigma_{A}^{2}+\sigma_{B}^{2}+\mu_{B}^{2}-\mu_{B}^{2}{\left(2p_{1}-1\right)}^{2}+\frac{\sigma^{2}}{T-m}.$$

The above expressions account for population-level information about the treatments through the program parameter $p_{1}$. For example, if emerging evidence in the literature suggests slight advantage of $x_{i}=1$ over $x_{i}=-1$, we may assume the physicians in the program will select $x_{i}\prime =1$ with $p_{1}>0.5$. In Appendix C, we provide expressions analogous to (4.6)–(4.8) for the situations where the physician may prescribe treatment $x_{i}\prime$ with patient-specific knowledge in addition to the population-level parameter $p_{1}$. However, we note that using expressions (4.6)–(4.8) may adequately reflect the standard of care where treatments are chosen based on population-level information rather than patient-specific knowledge. Furthermore, under the independence assumption of $x_{i}\prime$ and $\beta_{i}$, the power expression depends only on model parameters $\left(\sigma_{A},\sigma_{B},\mu_{B},\sigma ,\tau_{i}\right)$ for which information may be available to provide preliminary estimates and the known design parameters $\left(p_{1},m,n,T\right)$. Finally, under the null case $\mu_{B}=0$:

#### Main Result 2.

All else being equal, the power to demonstrate quality improvement due to N-of-1 trials (vs SOC) increases as heterogeneity of treatment effects $\sigma_{B}^{2}$ increases.

## 5. Numerical Illustrations: Application to ALS Patients

### 5.1. Optimal Length of Experimentation

**Figure 2.**
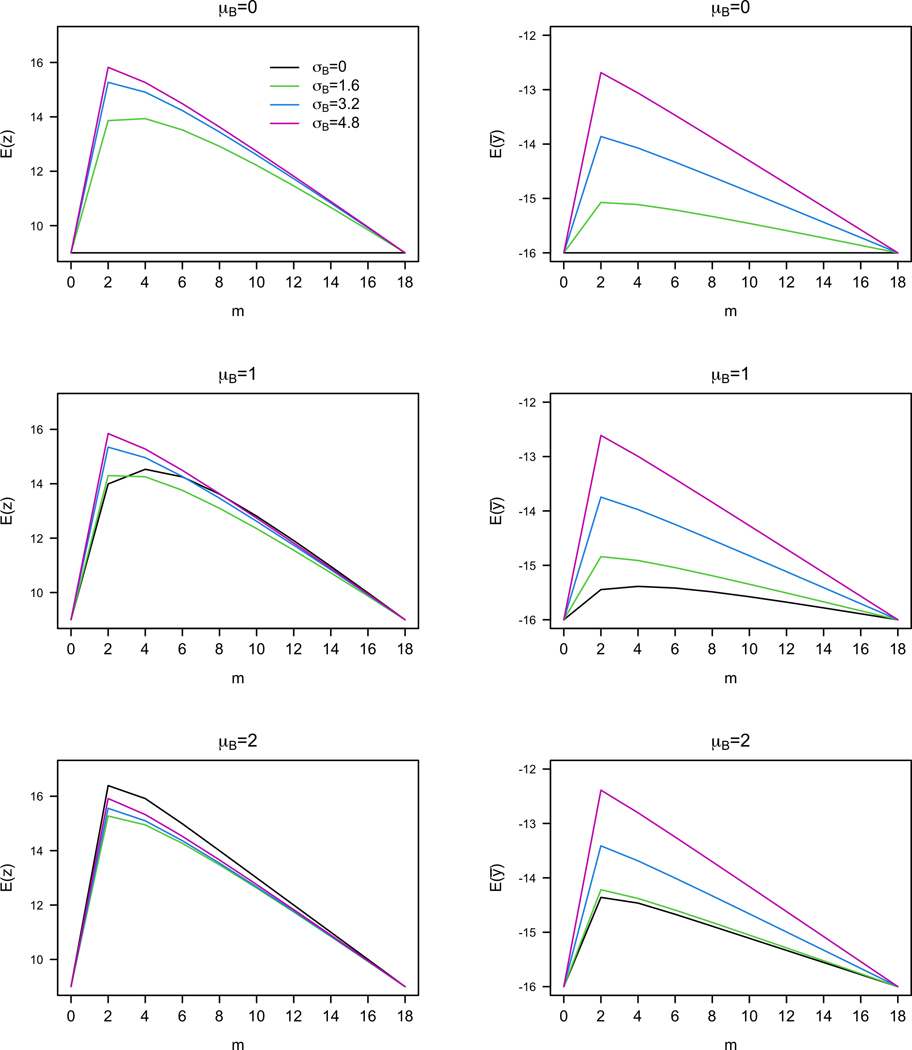
Patient-centric criteria vs experimentation length m under different values of $\mu_{B}$ and $\sigma_{B}$. Left: Expected number of optimal treatment periods vs m. Right: Expected average outcome (negative of MCS) of patient vs m.

We use the MCS natural history data in (Mitsumoto et al. 2019) to inform the design of the evaluation program for people with ALS. Specifically, we fitted a random effects model to the data and obtained an estimate of $\sigma_{A}=4.8$ and σ=1.6. For simplicity in illustration, we further assume that the within-subject noise is conditionally independent given the population-level parameters. Figure 2 plots the patient-centric criteria against different values of $\mu_{B},\sigma_{B},$ and m for T=18. While the two criteria adopt different metrics, they are maximized when m is relatively small. In Figure 2 and in all $\left(\mu_{B},\sigma_{B}\right)$ that we have considered (not shown here), the optimal values of m range from 2 to 6 for both criteria. This is consistent with what Main Result 1 implies: $m^{*}≲T/3=6$.

### 5.2. Sample Size and Effect Size

**Figure 3.**
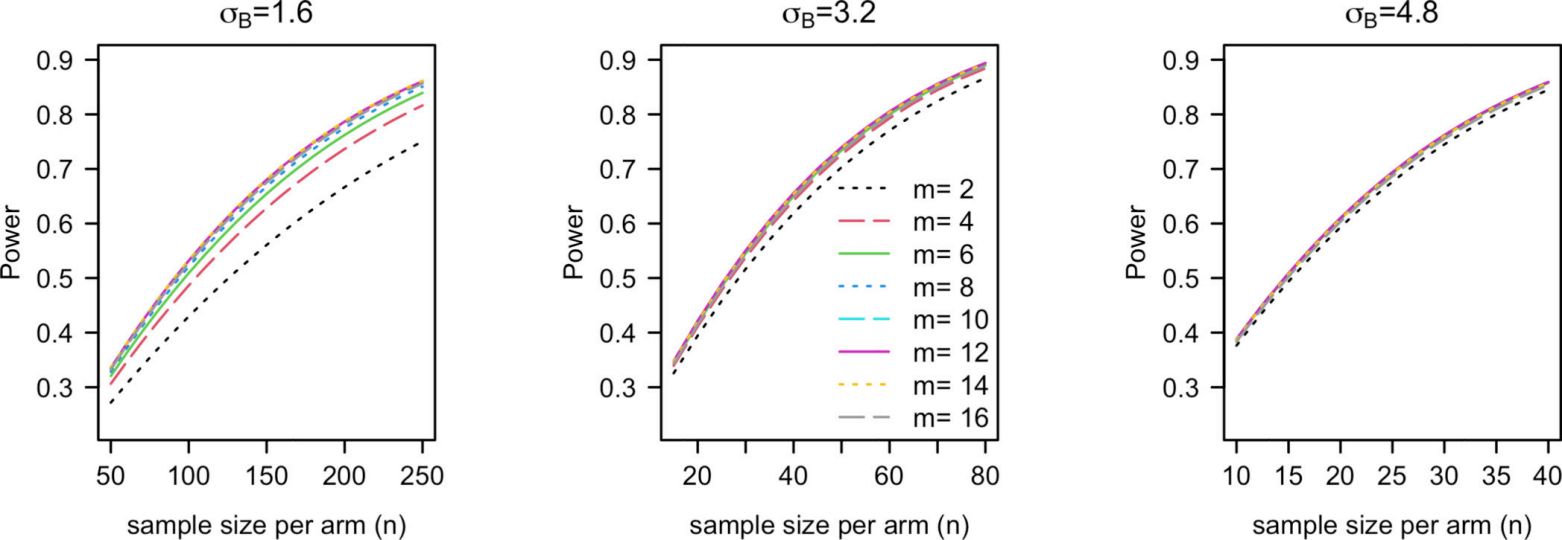
Power vs (n,m) for different values of $\sigma_{B}$ with $\mu_{B}=0$, $\sigma_{A}=4.8$, σ=1.6, ρ=0, and T=18.

Main Result 2 implies that $\sigma_{B}^{2}$ may be viewed as an effect size in power calculation, while the power also depends on other model parameters and design parameters. As in conventional practice, the choice of an effect size should be based on a clinically meaningful difference, whereas the other model parameters (e.g., $\sigma_{A},\sigma$, etc) may be based on pilot data if available. Figure 3 plots the power against (n,m) for three different effect sizes $\sigma_{B}$ for a one-sided test at 5% significance. Under each effect size, we identify the smallest n that achieves 80% power for any m and obtain that the required (n,m) are (210,12),(60,6), and (34,4) respectively for $\sigma_{B}=1.6,3.2,$ and 4.8. We note that under a small effect size $\sigma_{B}=1.6$, the required m=12 is greater than T/3. In light of Main Result 1, we may instead adopt (n,m)=(210,6) in order to maximize the benefits of the N-of-1 trials to the patients. The power of this modified design is 78%, which is slightly lower than the target 80%. Generally, we observe from Figure 3 that the impact of m on power is relatively small compared to that of n and $\sigma_{B}$ except when the effect size is small ($\sigma_{B}=1.6$).

To determine if a specific value of $\sigma_{B}$ corresponds to a clinically meaningful effect size, relating $\sigma_{B}$ to Δ using (4.6) may be useful, as Δ lives on the same scale as the measurement outcomes. In our application, a 3- to 4-point change on the MCS will represent a clinical meaningful shift. Based on the pilot data and assumptions, the effect size $\sigma_{B}=1.6,3.2,4.8$ translate to a degree of quality improvement Δ=1.2,2.5,3.8 respectively. Thus, we set the sample size for this evaluation program at n=34 with four treatment periods (two on mexiletine and two on baclofen) based on the results for $\sigma_{B}=4.8$. Generally, the minimally clinically important heterogeneity (MCIH $\sigma_{B,min}$) may be determined relating to the minimally clinically important change (MCID, $\Delta_{min}$) using (4.6).

### 5.3. Power for Comparing to Fully Informed SOC

The calculations in the previous subsection assume the null case $\mu_{B}=0$ under which the power (4.5) does not depend on the parameter $p_{1}$. Under a non-null case, the value of $p_{1}$ reflects how informed the practice is about the population-level treatment effect. For example, if evidence in the literature suggests $\mu_{B}>0$, an informed practice will prescribe $x_{i}\prime =1$ with $p_{1}>0.5$. Specifically, standard of care that is fully informed by the literature may correspond to $p_{1}=\text{Pr}(\beta_{i}>0)=\Phi (\mu_{B}/\sigma_{B})$.

Table 1 shows that as the average treatment effect $\mu_{B}$ grows larger and a fully informed SOC practice prescribes $x_{i}\prime =1$ more often (i.e., larger $p_{1}$), the power to demonstrate quality improvement in (4.3) becomes smaller. On the one hand, this suggests that if there is overwhelming evidence favoring $x_{i}\prime =1$ over $x_{i}\prime =-1$ in the literature, conducting N-of-1 trials will have diminished effect *provided that* the standard of care is fully informed. On the other hand, even with a large average treatment effect $\mu_{B}=2.4=0.5\sigma_{B}$, quality improvement due to N-of-1 trials Δ>3, which is still clinically meaningful and the power is still reasonable high (68%) in this sensitivity analysis. This suggests that evaluating N-of-1 trials is a worthwhile endeavor unless there is overwhelming evidence of a large average treatment effect.

The numerical results in this article, and power and the patient-centric criteria in general, can be computed using tools are available at: https://roadmap2health.io/hdsr/n1power/.

## 6. Discussion

N-of-1 trials have been increasingly used as a design tool to bridge practice and science in rare diseases (Müller et al. 2021)(n.d.b). However, the literature is missing concrete guidelines on N-of-1 designs as to how much experimentation is appropriate. A fundamental issue is the articulation of a framework that will facilitate the evaluation of the usefulness of N-of-1 trials. In this article, we introduce an evaluation framework and outline the basic elements in an evaluation program for N-of-1 trials—namely, an experimentation phase, a feedback period, and a validation phase. In the literature, the reporting of N-of-1 trials mostly focuses only on the results of the experimentation phase, where patients explore the different treatments sequentially under a rigorous clinical protocol such as randomization, blinding, and scheduled follow-up. The feedback period and the validation phase are the critical elements in the planning and the conduct of N-of-1 trials but are, unfortunately, often omitted in the description of the design and the analytical plan.

Specifically, the length of the validation phase, relative to that of the experimentation phase, should be given careful consideration. We have demonstrated theoretically and numerically that the optimal length of experimentation from the patient’s perspective should be no greater than one-third of the entire study duration. This implies a relatively long validation phase, suggesting the importance of reproducing the quality of the decisions due to N-of-1 trials with additional follow-up. Our theoretical results also provide guidance on how many patients are needed in order to adequately power for testing quality improvement. Importantly, the relative length of experimentation and validation has minimal impact on the power. In other words, little conflict exists between the goal of maximizing patient benefits and maximizing power.

The feedback period facilitates evidence-based treatment decisions using data measured in the experimentation phase. Summarizing the relative benefits of the treatments via a single numerical statistic is a pragmatic way to present such evidence, because the information can be objectively presented and quickly digested by stakeholders. We have developed design calculus based on the model-based least squares estimation, which is quick to compute and produces unbiased estimates of patient-specific treatment effects under a broad range of scenarios. Other more sophisticated model-based methods may be used to deal with the more complex situations. For example, when we observe high volume of outcome measures via wearable devices, we could extend model (3.2) to an autoregressive model with multiple observations per treatment period (Kronish et al. 2019). In practice, treatment decisions are likely determined based on the totality of evidence. For example, in situations where a treatment that apparently benefits a patient may have side effects, a possibly less effective treatment may be preferred if it is more easily tolerated. Considerations of multiple outcomes in the analysis during the feedback period will likely increase adherence and will warrant further empirical, domain-specific research. Overall, as the feedback period potentially changes the treatment decisions—and hence, the outcomes—in the validation phase, it can be viewed as an integral part of the intervention component. We may thus experiment in a randomized fashion different elements in the feedback period for different individuals: we may consider presenting different endpoints (e.g., muscle cramp or safety), using a single endpoint, a composite outcome, or multivariate endpoints, using different types of analyses (e.g., intent-to-treat vs per-protocol), and asking patients for their satisfaction and preference (Cheung et al. 2020).

Some considerations, assumptions, and limitations for power calculation in conventional randomized controlled trials also apply for N-of-1 trials. First, power calculation involves the inputs of a number of nuisance model parameters (e.g., $\sigma_{A},\sigma$) as well as the effect size ($\sigma_{B}^{2}$). While the effect size $\sigma_{B}^{2}$ should be determined based on clinical relevance shift, the other parameters ideally can be based on estimates from pilot data. However, in situations where robust pilot data are not available, a potential useful strategy is to leverage the concepts of adaptive designs (U.S. Food & Drug Administration 2019) whereby the model parameters are updated using interim data in the evaluation program and the updates in turn inform a reassessment of the degree of quality improvement and the sample size required.

Second, our derivations assume that patients in both arms comply with their treatments in the following sense: patients in the N-of-1 trials adhere to the estimated optimal treatments based on the experimentation phase data, and patients in the SOC continue with the same treatment as in the experimentation phase. If there is prior information about noncompliance rate, power expressions can be derived accordingly under the proposed framework. However, from the viewpoint that the feedback period is part of the N-of-1 trial intervention, it should be designed to maximize adherence by choosing the outcomes and analyses that most reflect patient preference as discussed in the previous paragraph. Third, approaches to deal with missing data should be prespecified and implemented during the feedback period. An advantage of using model-based estimation is that the model can also serve as the basis for multiple imputations. That being said, no statistical approach can replace a well-conducted trial that is characterized by good compliance to treatment and minimal missing data.

## Figures and Tables

**Table 1.** Quality improvement Δ and power for comparing to a fully informed SOC (standard of care) with $p_{1}=\Phi (\mu_{B}/\sigma_{B})$ with n=34,, m=4, T=18, $\sigma_{A}=4.8,$ $\sigma_{B}=4.8,\;\sigma =1.6$ and ρ=0.

$\mu_{B}$	$p_{1}=\Phi (\mu_{B}/\sigma_{B})$	Δ	Power
0	0.50	3.8	80%
1.2	0.60	3.7	77%
1.6	0.63	3.6	75%
2.4	0.69	3.3	68%
4.8	0.84	2.3	39%

## Appendices

### Appendix A. Theoretical Results Concerning $E(z_{i})$

#### A.1. Proof of Proposition 1.

First, consider the case $\beta_{i}>0$ for patient i so the optimal treatment is $x_{it}=1$. Then, the number of periods the patient is on the optimal treatment equals

(A.1)
$$z_{i}=\frac{m}{2}+(T-m)I(x_{i}^{*}=1)=\frac{m}{2}+(T-m)I({\hat{\beta }}_{i}>0).$$

The first term in the right-hand-side of (A.1) is the number of optimal treatment periods received in the experimental phase, and the second term is the number in the validation phase. Since ${\hat{\beta }}_{i}∼N(\beta_{i},\tau_{i}^{2})$, we have

$$E\{I({\hat{\beta }}_{i}>0)|\alpha_{i},\beta_{i}\}=\text{Pr}({\hat{\beta }}_{i}>0|\alpha_{i},\beta_{i})=\Phi (\beta_{i}/\tau_{i}),$$

and therefore,

(A.2)
$$E(z_{i}|\alpha_{i},\beta_{i})=\frac{m}{2}+(T-m)\Phi (\beta_{i}/\tau_{i})\;\text{when}\;\beta_{i}>0.$$

Next, under the case $\beta_{i}<0$, we can analogously derive that

(A.3)
$$E(z_{i}|\alpha_{i},\beta_{i})=\frac{m}{2}+(T-m)\Phi (-\beta_{i}/\tau_{i})\;\text{when}\;\beta_{i}<0.$$

Combining (A.2) and (A.3) gives

(A.4)
$$E(z_{i}|\alpha_{i},\beta_{i})=E(z_{i}|\beta_{i})=\frac{m}{2}+(T-m)\Phi (|\beta_{i}|/\tau_{i}),$$

which is free of $\alpha_{i}$. Since $E(z_{i})=E\{E(z_{i}|\beta_{i})\}$, the expectations of both sides in (A.4) are to be taken with respect to the distribution of $\beta_{i}∼N(\mu_{B},\sigma_{B}^{2})$ to complete the proof. By change of variable, we have

(A.5)
$$E\;\Phi (|\beta_{i}|/\tau_{i})=\int_{-\infty }^{\infty }\int_{-\infty }^{\frac{\left|b\right|}{\tau_{i}}}\frac{1}{\sigma_{B}}\phi (w)\phi (\frac{b-\mu_{B}}{\sigma_{B}})dwdb\;\;=\int_{-\infty }^{\infty }\int_{-\infty }^{\frac{\left|\mu_{B}+\sigma_{B}u\right|}{\tau_{i}}}\phi (w)\phi (u)dwdu\;\;=\text{Pr}(W\leq \frac{\left|\mu_{B}+\sigma_{B}U\right|}{\tau_{i}}).$$

The proof is completed by substituting (A.5) into (A.4).

### Appendix B. Theoretical Results Concerning $E({\overset{¯}{y}}_{i})$

#### B.1. Lemma 1

Derivations of $E({\overset{¯}{y}}_{i})$ will be facilitated by first noting the following lemma:

##### Lemma 1.

*Let* $V∼N(\mu_{V},\sigma_{V}^{2})$. *Then*,

$$E\{V\Phi (V)\}=\mu_{V}\Phi (\frac{\mu_{V}}{\sqrt{\sigma_{V}^{2}+1}})+\frac{\sigma_{V}^{2}}{\sqrt{\sigma_{V}^{2}+1}}\phi (\frac{\mu_{V}}{\sqrt{\sigma_{V}^{2}+1}})$$

*where* Φ *and* ϕ *respectively denote the standard normal distribution function and density function*.

##### Proof of Lemma 1:

Using definition of expectation, we derive

(B.1)
$$E\{V\Phi (V)\}=\int_{-\infty }^{\infty }\int_{-\infty }^{v}v\phi (u)\frac{1}{\sigma_{V}}\phi (\frac{v-\mu_{V}}{\sigma_{V}})dudv\;\;=\int_{-\infty }^{\infty }\int_{-\infty }^{\mu_{V}+\sigma_{V}w}\left(\mu_{V}+\sigma_{V}w\right)\phi (u)\phi (w)dudw\;\;=\mu_{V}\text{Pr}(U<\mu_{V}+\sigma_{V}W)+\sigma_{V}\int_{-\infty }^{\infty }w\Phi (\mu_{V}+\sigma_{V}w)\phi (w)dw\;\;=\mu_{V}\Phi (\frac{\mu_{V}}{\sqrt{1+\sigma_{V}^{2}}})+\sigma_{V}^{2}\int_{-\infty }^{\infty }\phi (w)\phi (\mu_{V}+\sigma_{V}w)dw$$

where U,W are independent standard normal variables. Thus, $U-\sigma_{V}W∼N(0,1+\sigma_{V}^{2})$, and the first term in (B.1) can be evaluated as

(B.2)
$$\mu_{V}\text{Pr}(U<\mu_{V}+\sigma_{V}W)=\mu_{V}\Phi (\frac{\mu_{V}}{\sqrt{1+\sigma_{V}^{2}}}).$$

Next, the single integral in the second term in (B.2) can be evaluated using integration by parts

(B.3)
$$\int_{-\infty }^{\infty }w\Phi (\mu_{V}+\sigma_{V}w)\phi (w)dw=\sigma_{V}\int_{-\infty }^{\infty }\phi (\mu_{V}+\sigma_{V}w)\phi (w)dw\;\;=\sigma_{V}\frac{1}{\sqrt{\sigma_{V}^{2}+1}}\phi (\frac{\mu_{V}}{\sqrt{\sigma_{V}^{2}+1}}).$$

Equation (B.3) can be derived by straightforward derivation. The proof of Lemma 1 is thus completed by plugging (B.2) and (B.3) into (B.1).

#### B.2. Proof of Proposition 2

Recall that ${\overset{¯}{y}}_{i}$ denotes the average outcome of patient i in all T treatment periods in an N-of-1 trial. Hence,

(B.4)
$$E({\overset{¯}{y}}_{i})=\frac{1}{T}\sum_{t=1}^{T}E(\alpha_{i}+\beta_{i}x_{it}+\epsilon_{it})=\mu_{A}+\frac{1}{T}\sum_{t=1}^{T}E(\beta_{i}x_{it})\;\;=\mu_{A}+(1-\frac{m}{T})E(\beta_{i}x_{i}^{*}).$$

Equation (B.4) holds as because of balanced design $\sum_{i=1}^{m}x_{it}=0$. Next, since ${\hat{\beta }}_{i}∼N(\beta_{i},\tau_{i}^{2})$, we have

(B.5)
$$E(\beta_{i}x_{i}^{*})=E\{\beta_{i}E(x_{i}^{*}|\beta_{i})\}=E[\beta_{i}E\{2I({\hat{\beta }}_{i}>0)-1|\beta_{i}\}]=E\{2\beta_{i}\Phi (\frac{\beta_{i}}{\tau_{i}})-\beta_{i}\}\;\;=2E\{\beta_{i}\Phi (\frac{\sqrt{m}\beta_{i}}{\sigma })\}-\mu_{B}\;\;=2\tau_{i}E\{\frac{\beta_{i}}{\tau_{i}}\Phi (\frac{\beta_{i}}{\tau_{i}})\}-\mu_{B}\;\;=\mu_{B}\{2\Phi (\frac{\mu_{B}}{\sqrt{\sigma_{B}^{2}+\tau_{i}^{2}}})-1\}+\frac{2\sigma_{B}^{2}}{\sqrt{\sigma_{B}^{2}+\tau_{i}^{2}}}\phi (\frac{\mu_{B}}{\sqrt{\sigma_{B}^{2}+\tau_{i}^{2}}}).$$

Expression (B.5) is obtained by applying Lemma 1 with $V=\beta_{i}/\tau_{i}$. Putting (B.5) into (B.4) gives

(B.6)
$$E({\overset{¯}{y}}_{i})=\mu_{A}+(1-\frac{m}{T})[\mu_{B}\{2\Phi (\frac{\mu_{B}}{\sqrt{\sigma_{B}^{2}+\tau_{i}^{2}}})-1\}+\frac{2\sigma_{B}^{2}}{\sqrt{\sigma_{B}^{2}+\tau_{i}^{2}}}\phi (\frac{\mu_{B}}{\sqrt{\sigma_{B}^{2}+\tau_{i}^{2}}})]$$

thus completing the proof of Proposition 2.

#### B.3. Derivation of optimal experimentation length $m^{*}$ and Main Result 1

For least squares ${\hat{\beta }}_{i}^{LS}$, the variance $\tau_{i}^{2}=\lambda_{i}\sigma^{2}/m$, where $\lambda_{i}=1+\sum_{s\neq t}x_{is}x_{it}\rho_{st}/m$. Further supposing $\mu_{B}=0$ simplifies (B.6) to

$$E({\overset{¯}{y}}_{i})=\mu_{A}+(1-\frac{m}{T})\frac{2\sigma_{B}^{2}}{\sqrt{\sigma_{B}^{2}+\lambda_{i}\sigma^{2}/m}}\phi (0).$$

Hence, maximizing $E({\overset{¯}{y}}_{i})$ as a function of m is equivalent to maximizing the function

$$h(m)=(1-\frac{m}{T})\frac{1}{\sqrt{\xi_{i}+1/m}}$$

where $\xi_{i}=\sigma_{B}^{2}/(\lambda_{i}\sigma^{2})$ is free of m. Using standard calculus arguments, we can show that the maximizer $m^{*}$ of h(m) solves the equation $2\xi_{i}m^{*2}+3m^{*}-T=0$ or equivalently,

(B.7)
$$m^{*}=\frac{\sqrt{9+8\xi_{i}T}-3}{4\xi_{i}}.$$

The derivation of $m^{*}$ is completed by multiplying $\sqrt{9+8\xi_{i}}+3$ in the numerator and the denominator of (B.7), which gives

$$m^{*}=\frac{2T}{\sqrt{9+8\xi_{i}T}+3}.$$

Now, since $\xi_{i}\geq 0$, we have $m^{*}\leq 2T/\sqrt{9}+3=T/3$. As a practical note, due to discreteness in m, the optimal m may be a result of rounding up $m^{*}$. Hence a slightly less sharp inequality would be $m^{*}≲T/3<T/3+1$.

### Appendix C. Theoretical Results Concerning Power

In this section, we derive the expressions involved in the power of the Z-test—namely, Δ, var $\left(y_{i}^{*}\right)$, and var $\left(y_{i}\prime \right)$.

Recall that $p_{0}$ and $p_{1}$ respectively denote the probabilities that the treating physicians will prescribe mexiletine $\left(x_{it}=-1\right)$ and baclofen $\left(x_{it}=1\right)$ under the treatment program. Based on model (3.2), we can express the quality of an N-of-1 trial as:

$$\begin{array}{rrrrr} E(y_{i}^{*}) & = & \frac{1}{T-m}\sum_{t=m+1}^{T}E(\alpha_{i}+\beta_{i}x_{it}+\epsilon_{it}) & =\frac{1}{T-m}\sum_{t=m+1}^{T}E(\alpha_{i}+\beta_{i}x_{i}^{*}+\epsilon_{it}) & =\mu_{A}+E(\beta_{i}x_{i}^{*}). \end{array}$$

and analogously $E(y_{i}\prime )=\mu_{A}+E(\beta_{i}x_{i}\prime )$ where $x_{i}\prime$ is the treatment given to patient i in SOC. Hence $\Delta =E(\beta_{i}x_{i}^{*})-E(\beta_{i}x_{i}\prime )$. Under the independence assumption of $x_{i}\prime$ and $\beta_{i}$, we further obtain $E(y_{i}\prime )=\mu_{A}+(2p_{1}-1)\mu_{B}$, and

(C.1)
$$\Delta =E(\beta_{i}x_{i}^{*})-\mu_{B}(2p_{1}-1)\;\;=\mu_{B}\{2\Phi (\frac{\mu_{B}}{\sqrt{\sigma_{B}^{2}+\tau_{i}^{2}}})-1\}+\frac{2\sigma_{B}^{2}}{\sqrt{\sigma_{B}^{2}+\tau_{i}^{2}}}\phi (\frac{\mu_{B}}{\sqrt{\sigma_{B}^{2}+\tau_{i}^{2}}})-\mu_{B}(2p_{1}-1)\;\;=2\mu_{B}\{\Phi (\frac{\mu_{B}}{\sqrt{\sigma_{B}^{2}+\tau_{i}^{2}}})-p_{1}\}+\frac{2\sigma_{B}^{2}}{\sqrt{\sigma_{B}^{2}+\tau_{i}^{2}}}\phi (\frac{\mu_{B}}{\sqrt{\sigma_{B}^{2}+\tau_{i}^{2}}})$$

where $E(\beta_{i}x_{i}^{*})$ is given in (B.5).

Next,

$$\text{var}(y_{i}^{*})=\text{var}\{\alpha_{i}+\beta_{i}x_{i}^{*}+\sum_{t=m+1}^{T}\epsilon_{it}/(T-m)\}\;\;=\sigma_{A}^{2}+\text{var}(\beta_{i}x_{i}^{*})+\frac{\sigma^{2}}{T-m}=\sigma_{A}^{2}+\sigma_{B}^{2}+\mu_{B}^{2}-\{E(\beta_{i}x_{i}^{*}){\}}^{2}+\frac{\sigma^{2}}{T-m}\;\;=\sigma_{A}^{2}+\sigma_{B}^{2}+\mu_{B}^{2}-\{\Delta +\mu_{B}(2p_{1}-1){\}}^{2}+\frac{\sigma^{2}}{T-m}.:=\sigma^{*2}$$

The last equality is a result of (C.1). Similarly, we can show

$$\text{var}(y_{i}\prime )=\sigma_{A}^{2}+\sigma_{B}^{2}+\mu_{B}^{2}-\mu_{B}^{2}{\left(Ex_{i}\prime \right)}^{2}+\frac{\sigma^{2}}{T-m}\;\;=\sigma_{A}^{2}+\sigma_{B}^{2}+\mu_{B}^{2}-\mu_{B}^{2}{\left(2p_{1}-1\right)}^{2}+\frac{\sigma^{2}}{T-m}.$$

Finally, under the null $\mu_{B}=0$, we have

$$\frac{\Delta }{\sqrt{\text{var}(y_{i}^{*})+\text{var}(y_{i}\prime )}}=\frac{2\sigma_{B}^{2}\phi (0)}{\sqrt{\sigma_{B}^{2}+\tau_{i}^{2}}\sqrt{2\sigma_{A}^{2}+2\sigma_{B}^{2}-\frac{4\sigma_{B}^{4}\phi^{2}(0)}{\sigma_{B}^{2}+\tau_{i}^{2}}+2\sigma^{2}/(T-m)}}.$$

Main Result 2 is proved by dividing $\sigma_{B}^{2}$ on the numerator and the denominator in the above expression, as a result of which the numerator will be a constant and the denominator will be a decreasing function $\sigma_{B}^{2}$.

For the situations where the physicians have patient-specific knowledge to inform treatments under the SOC, we may postulate that

(C.2)
$$x_{i}\prime =\{\begin{array}{ll} 2I(\beta_{i}>0)-1 & \text{with probability}\;\theta_{C}\\ \;1 & \text{with probability}\;(1-\theta_{C})p_{1}\\ \;-1 & \text{with probability}\;(1-\theta_{C})(1-p_{1}). \end{array}$$

The parameter $\theta_{C}$ indicates how perfect the knowledge the physicians have about the specific best treatments for their patients, with $\theta_{C}=1$ indicating perfect knowledge and $\theta_{C}=0$ indicating no additional knowledge beyond the population-level information $p_{1}$. Under the SOC treatment system (C.2), we have

(C.3)
$$E(\beta_{i}x_{i}\prime )=E\{\beta_{i}E(x_{i}\prime |\beta_{i})\}=2\theta_{C}E\{\beta_{i}I(\beta_{i}>0\}-\theta_{C}\mu_{B}+(1-\theta_{C})\mu_{B}(2p_{1}-1)$$

where

(C.4)
$$E\{\beta_{i}I(\beta_{i}>0\}=\sigma_{B}\phi (\mu_{B}/\sigma_{B})+\mu_{B}\Phi (\mu_{B}/\sigma_{B}).$$

Using (B.5), (C.3), and (C.4), after some algebra, we have

$$\Delta =E(\beta_{i}x_{i}^{*})-E(\beta_{i}x_{i}\prime )\;\;=2(1-\theta_{C})\mu_{B}\{\Phi (\frac{\mu_{B}}{\sqrt{\sigma_{B}^{2}+\tau_{i}^{2}}})-p_{1}\}+\frac{2(1-\theta_{C})\sigma_{B}^{2}}{\sqrt{\sigma_{B}^{2}+\tau_{i}^{2}}}\phi (\frac{\mu_{B}}{\sqrt{\sigma_{B}^{2}+\tau_{i}^{2}}})+\;\;2\theta_{C}\mu_{B}\{\Phi (\frac{\mu_{B}}{\sqrt{\sigma_{B}^{2}+\tau_{i}^{2}}})-\Phi (\frac{\mu_{B}}{\sigma_{B}})\}+2\theta_{C}[\frac{\sigma_{B}^{2}}{\sqrt{\sigma_{B}^{2}+\tau_{i}^{2}}}\phi (\frac{\mu_{B}}{\sqrt{\sigma_{B}^{2}+\tau_{i}^{2}}})-\sigma_{B}\phi (\frac{\mu_{B}}{\sigma_{B}})]$$

It is instructive to consider the null case $\mu_{B}=0$, under which

$$\Delta =\frac{2\sigma_{B}^{2}\phi (0)}{\sqrt{\sigma_{B}^{2}+\tau_{i}^{2}}}-2\theta_{C}\sigma_{B}\phi (0).$$

Hence, quality improvement Δ due to N-of-1 trials diminishes as the standard of care (SOC) patient-specific knowledge $\theta_{C}$ increases. Also under this special case, Δ>0 if and only if $\theta_{C}^{2}<\sigma_{B}^{2}/(\sigma_{B}^{2}+\tau_{i}^{2})$

Similarly, we can obtain $\text{var}(y_{i}^{*})$ and $\text{var}(y_{i}\prime )$ under the SOC treatment system (C.2) by plugging (B.5) and (C.3) respectively in the followings:

$$\text{var}(y_{i}^{*})=\sigma_{A}^{2}+\sigma_{B}^{2}+\mu_{B}^{2}-{\left\{E(\beta_{i}x_{i}^{*})\right\}}^{2}+\frac{\sigma^{2}}{T-m}$$

and

$$\text{var}(y_{i}\prime )=\sigma_{A}^{2}+\sigma_{B}^{2}+\mu_{B}^{2}-{\left\{E(\beta_{i}x_{i}\prime )\right\}}^{2}+\frac{\sigma^{2}}{T-m}$$

Then under the null $\mu_{B}=0$, we have

$$\frac{\Delta }{\sqrt{\text{var}(y_{i}^{*})+\text{var}(y_{i}\prime )}}=\frac{2\sigma_{B}^{2}\phi (0)-2\theta_{C}\sigma_{B}\sqrt{\sigma_{B}^{2}+\tau_{i}^{2}}\phi (0)}{\sqrt{\sigma_{B}^{2}+\tau_{i}^{2}}\sqrt{2\sigma_{A}^{2}+2\sigma_{B}^{2}-\frac{4\sigma_{B}^{4}\phi^{2}(0)}{\sigma_{B}^{2}+\tau_{i}^{2}}-4\theta_{C}^{2}\sigma_{B}^{2}\phi^{2}(0)+2\sigma^{2}/(T-m)}}.$$

By dividing $\sigma_{B}^{2}$ on the numerator and the denominator in the above expression, we can see that the numerator is an increasing function in $\sigma_{B}^{2}$ and the denominator is a decreasing function in $\sigma_{B}^{2}$. Hence, Main Result 2 holds under this general case.
